# Supplementary material for: Precise inhibitory microcircuit assembly of developmentally related neocortical interneurons in clusters
Source: Nat Commun. 2017 Jul 13;8:16091. doi: 10.1038/ncomms16091 (PMC5511369; doi:10.1038/ncomms16091)
Supplement: Supplementary Information [file ncomms16091-s1.pdf]

File name: Supplementary Information
Description: Supplementary Figures, Supplementary Table and Supplementary References

File name: Supplementary Data 1
Description: Summary datasheets showing the spatial coordinates and the intra-clonal and inter-clonal Euclidean distances of the labeled cortical interneuron clones in the barcoded datasets by Mayer et al. and Harwell et al.

File name: Peer Review File
Description:

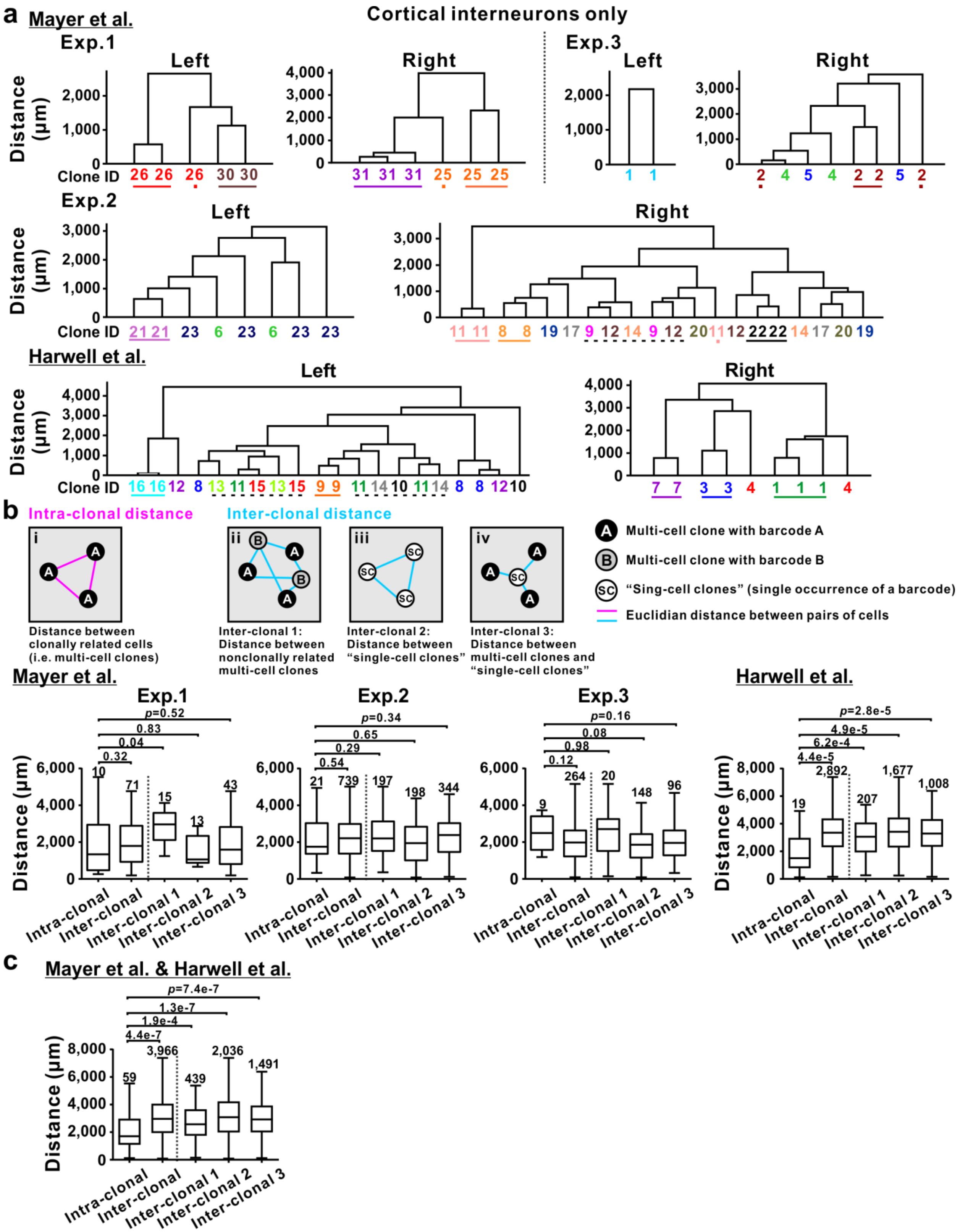

Supplementary Figure 1: Clonally related interneurons reliably form local clusters in the cortex. (a) Dendrograms of multi-cell clones in the left or right cortical hemisphere of the individual barcoded datasets with

single-cell resolution of clonal identity from Mayer et al. (n=3 brains)[1] and Harwell et al. (n=1 brain)[2]. Dendrograms were created by the linkage function according to their Euclidean distances (MATLAB, MathWorks). Numbers and colors indicate the lineal relationship between two or more cells based on the recovered barcodes. Colored lines below the numbers indicate spatially isolated clonal clusters. Colored dots mark a sibling neuron located away from the corresponding clonal cluster. Broken black lines indicate two local clonal clusters occupying the same or nearby space. **(b)** Quantifications of the average intra-clonal and inter-clonal Euclidean distances of all labeled cortical interneurons in individual brains of the Mayer et al. or Harwell et al. datasets as illustrated in the inset (top). The data are presented as the box and whisker plot with whiskers indicating the minimum and maximum values. The number above the whiskers indicates the total number of intra-clonal or inter-clonal interneuron pairs. The value above the lines indicates the statistical significance (Mann-Whitney t-test). Similar data display is used in the subsequent panel. **(c)** Quantifications of the average intra-clonal and inter-clonal Euclidean distances of all labeled cortical interneurons in all four brains of the Mayer et al. and Harwell et al. datasets as illustrated in *b*. Note that the intra-clonal distance is highly significantly shorter than the inter-clonal distances calculated in different ways, suggesting that spatial clustering is a reliable feature of clonally-related interneuron in the cortex. The value above the lines indicates the statistical significance (Mann-Whitney t-test).

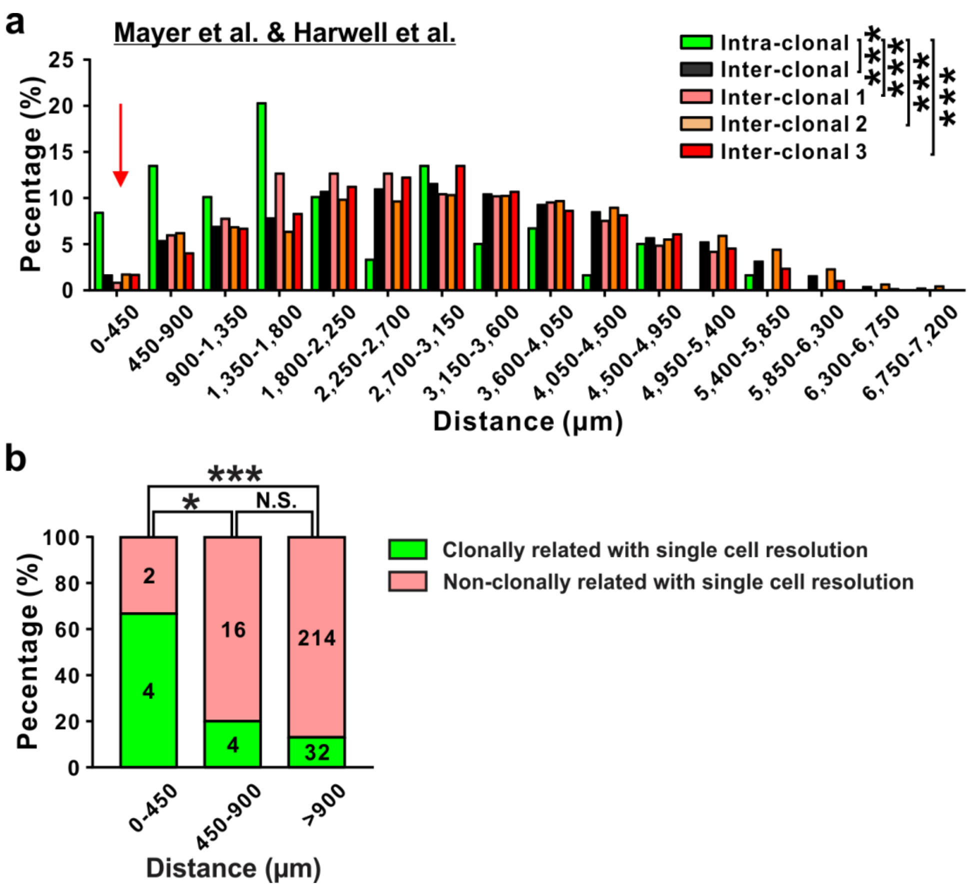

Supplementary Figure 2: Spatial clustering of clonally related interneurons is more prominent at the short distance range. (a) Histograms of the inter-clonal and intra-clonal Euclidean distances of all labeled cortical interneuron clones in Mayer et al. and Harwell et al. datasets (n=4 brains). ***, $p<0.001$ (Kolmogorov-Smirnov test). Red arrow indicates that within 0-450 μm distance range of our electrophysiological experiments, clonally related interneuron pairs (green) are more abundant than non-clonally related interneuron pairs (black). **(b)** Quantification of the percentage of sparsely labeled interneurons that are definitively clonally related in Mayer et al. at different distance ranges. Note that within 450 μm, 67% of labeled interneuron pairs are unequivocally clonally related. The numbers of intra-clonal or inter-clonal interneuron pairs within each distance range are listed in the bar graph. *, $p<0.05$; ***, $p<0.001$; N.S., not significant (chi-square test).

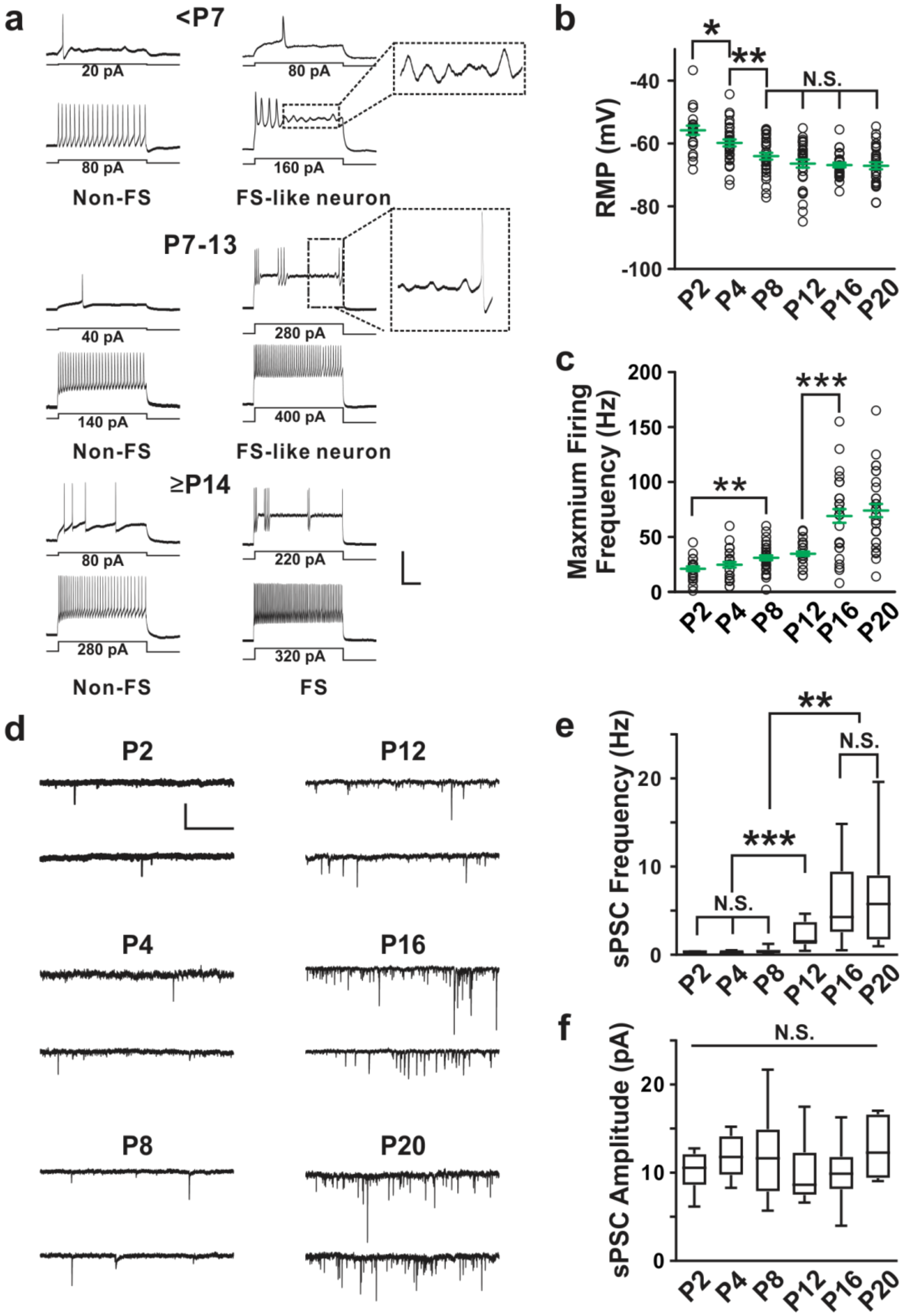

Supplementary Figure 3: Progressive maturation in membrane properties and synaptic activity of sparsely labeled neocortical interneurons in clusters. (a) Representative sample traces of the responses of EGFP-expressing interneurons arising from the MGE/PoA at E12 to somatic current injections at different postnatal time points. Subthreshold membrane potential oscillations, an early indication of FS interneuron, are shown in the insets. Scale bars: 50 mV and 200 msec. **(b)** Summary of the resting membrane potential (RMP) at different postnatal time points (P2, -55.8 ± 1.5 mV, n=22; P4, -59.9 ± 1.1 mV, n=31; P8, -64.1 ± 1.1 mV, n=32; P12, -66.4 ± 1.3 mV, n=32; P16, -66.9 ± 0.7 mV, n=29; P20, -67.1 ± 1.1 mV, n=30). Note a progressive hyperpolarization of the RMP as time

proceeds. Thick green lines indicate mean ± s.e.m.; *, $p<0.05$; **, $p<0.01$; N.S., not significant (unpaired t-test). **(c)** Summary of the maximum firing frequency at different postnatal time points (P2, 21.1 ± 2.1 Hz, n=22; P4, 24.8 ± 2.5 Hz, n=31; P8, 31.1 ± 2.1 Hz, n=32; P12, 34.8 ± 2.0 Hz, n=28; P16, 69.1 ± 6.3 Hz, n=30; P20, 74.1 ± 6.0 Hz, n=30). Note a progressive increase in the maximum firing frequency as time proceeds. Thick green lines indicate mean ± s.e.m.; **, $p<0.01$; ***, $p<0.001$ (unpaired t-test). **(d)** Sample traces of spontaneous postsynaptic currents (sPSCs) recorded from EGFP-expressing interneurons at different postnatal time points. Scale bars: 20 pA and 1 sec. **(e, f)** Summary of the frequency (e) (P2, 0.3 ± 0.03 Hz, n=9; P4, 0.3 ± 0.05 Hz, n=9; P8, 0.4 ± 0.1 Hz, n=14; P12, 2.1 ± 0.4 Hz, n=15; P16, 6.1 ± 1.4 Hz, n=11; P20, 6.0 ± 1.3 Hz, n=15) and amplitude (f) (P2, 10.3 ± 0.8 pA, n=9; P4, 11.7 ± 0.8 pA, n=9; P8, 12.3 ± 1.3 pA, n=14; P12, 10.2 ± 0.9 pA, n=15; P16, 10.0 ± 1.0 pA, n=11; P20, 12.7 ± 0.8 pA, n=15) of sPSCs at different postnatal time points. Note that the frequency of sPSCs drastically increased but plateaued after P14, whereas the average amplitude of sPSCs did not change significantly. The data are presented as the box and whisker plot with whiskers indicating the minimum and maximum values. **, $p<0.01$; ***, $p<0.001$; N.S., not significant (unpaired t-test).

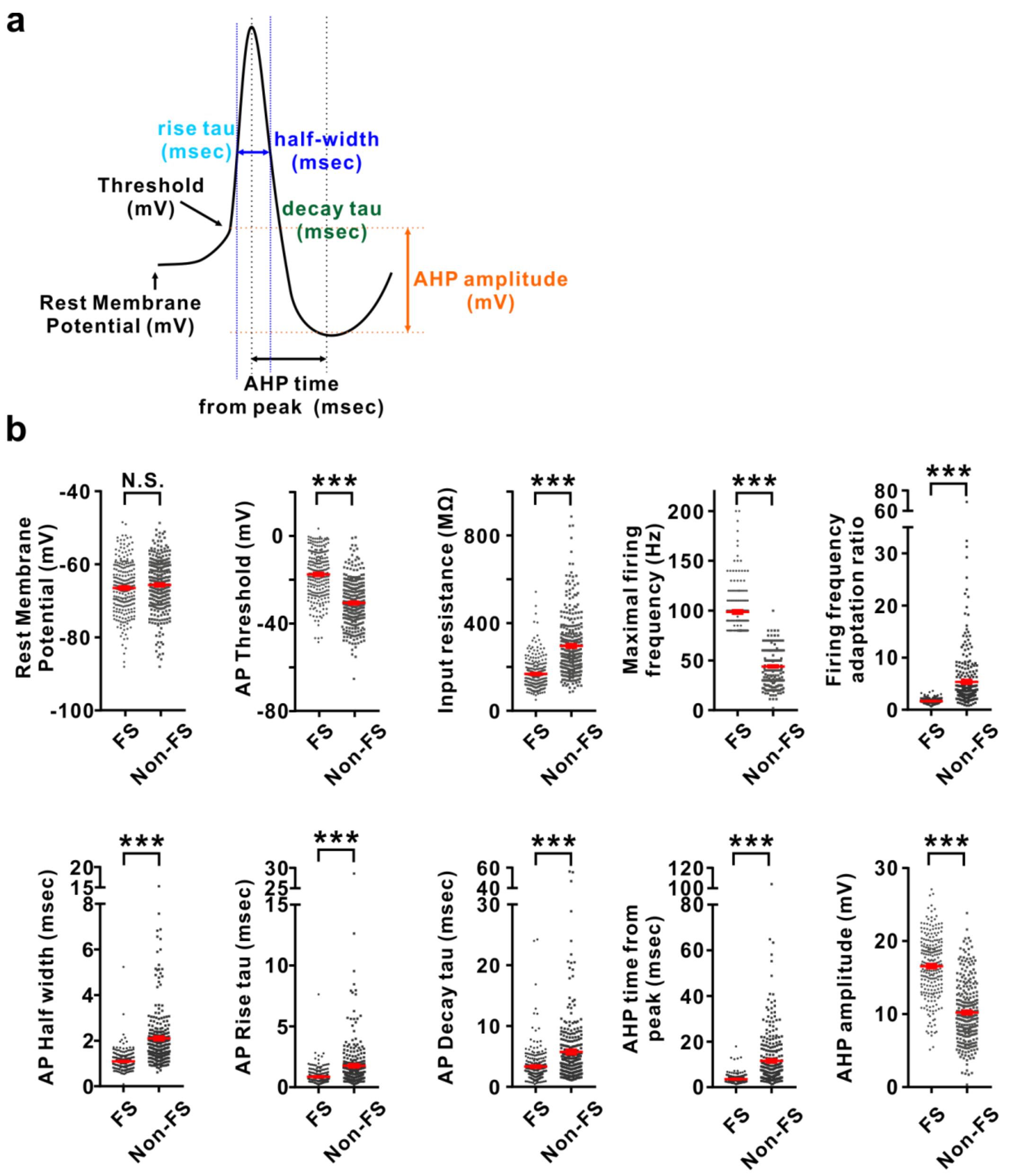

Supplementary Figure 4: Intrinsic membrane and firing properties of recorded neocortical FS and Non-FS interneurons. (a) Schematic diagram of an AP and related analyses. **(b)** Quantifications of the resting membrane potential, AP threshold, input resistance, maximal firing frequency, firing frequency adaptation ratio, AP half width, AP rise and delay time constants, AHP time from peak, AHP amplitude of the recorded FS (n=243) and Non-FS (n=292) interneurons (≥P14). Note that FS interneurons exhibited significantly higher AP threshold, AHP amplitude, and maximal firing frequency, but smaller input resistance, spike frequency adaptation, AP half-width, AP rise and decay time constants, and AHP time from peak, than non-FS interneurons. Individual dots and squares represent individual recorded interneurons. Thick red lines indicate mean ± s.e.m.; ***, p<0.001; N.S., not significant (Mann-Whitney t-test).

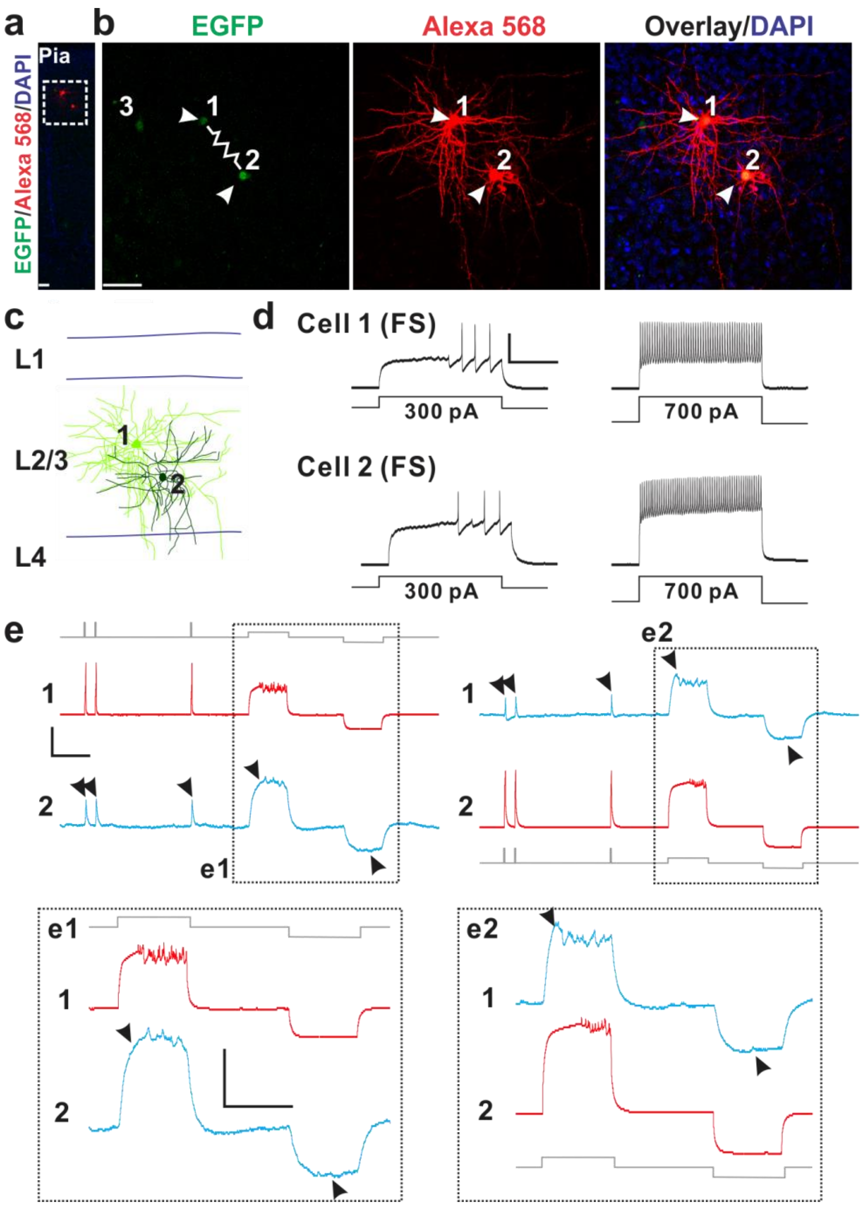

Supplementary Figure 5: Electrical synapse formation between sparsely labeled FS interneurons in clusters. (a, b) Confocal images of a pair of sparsely labeled EGFP-expressing interneurons (green, 1 and 2, broken lines and arrowheads) in cluster labeled at E12, examined by dual whole-cell patch clamp recordings at P14 and stained with DAPI (blue). Alexa 568 hydrazide (red) was included in the recording pipettes to confirm the identity of the recorded neurons. The wavy line indicates electrical coupling. Scale bar: 50 µm. **(c)** Morphological reconstruction of the two recorded sparsely labeled interneurons in *a*. **(d)** Firing patterns of the two sparsely labeled interneurons in *a*. Note that both cells are FS. Scale bars: 50 mV and 200 msec. **(e)** Dual whole-cell recordings of the two sparsely labeled interneurons in *a*. Brief and long duration depolarizing and hyperpolarizing current injections (gray) in one of the two sparsely labeled interneurons (driver; red) led to simultaneous depolarization or hyperpolarization of the

other labeled interneuron (receiver; blue, arrowheads), indicating the electrical coupling of the two sparsely labeled interneurons in cluster. Scale bars: 1,200 pA (gray), 50 mV (red), 5 mV (blue), and 200 msec.

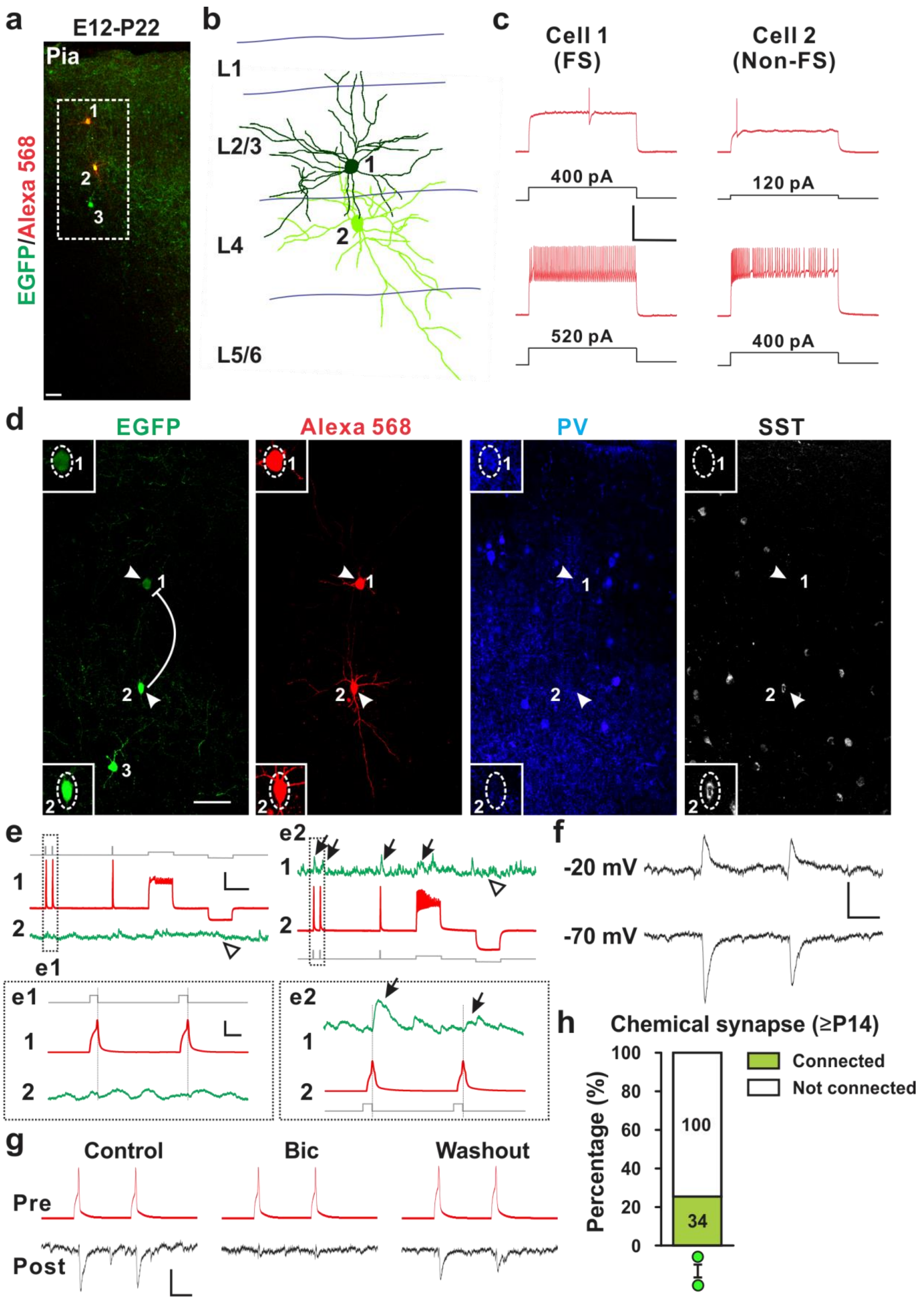

Supplementary Figure 6: Chemical synapse formation between sparsely labeled neocortical interneurons in clusters. (a) Confocal image of a pair of sparsely labeled EGFP-expressing interneurons (broken lines) in cluster by *in utero* intraventricular RCAS injection at E12 and examined by dual whole-cell patch clamp recordings at P22.

Alexa 568 hydrazide (red) was included in the recording pipettes to confirm the identity of the recorded neurons. Scale bar: 50 μm. **(b)** Morphological reconstruction of the two recorded sparsely labeled interneurons in *a*. **(c)** Firing patterns of the two recorded sparsely labeled interneurons in *a* responding to somatic current injections. Note that cell 1 is FS and cell 2 is Non-FS. Scale bars: 50 mV and 400 msec. **(d)** Confocal images of the two sparsely labeled interneurons expressing EGFP (green, 1 and 2, arrowheads) in *a* filled with Alexa 568 hydrazide (red) and stained for PV (blue) and SST (white). High magnification images of the cell bodies (broken lines) are shown in the insets. The bar-headed line indicates the chemical synaptic connection. Note that cell 1 is PV-positive whereas cell 2 is SST-positive. Scale bar: 50 μm. **(e)** Dual whole-cell recordings of the two sparsely labeled interneurons in *a*. Brief and long duration depolarizing currents (gray) were injected sequentially into one of the two sparsely labeled interneurons (presynaptic, red) to trigger action potentials and the postsynaptic responses were monitored in the other labeled interneuron (postsynaptic, green) under current-clamp mode. Note that action potentials in cell 2 reliably elicited postsynaptic responses in cell 1 (arrows) with a brief delay (broken lines). A similar panel layout is used in subsequent figures. Scale bars: 1,200 pA (gray), 40 mV (red), 0.5 mV (green), and 200 msec; 1,200 pA (gray), 50 mV (red), 0.2 mV (green), and 10 msec (insets). **(f)** Postsynaptic responses in cell 1 recorded at -70 mV and -20 mV under voltage-clamp mode. Note that the responses switch from inward to outward, consistent with the reversal potential (-35 mV) of the postsynaptic response. Scale bars: 20 pA and 20 msec. **(g)** Blockade of postsynaptic responses by the GABA-A receptor antagonist bicuculline (Bic, 100 μM). Scale bars: 50 mV (red), 20 pA (black), and 20 msec. **(h)** Summary of the frequency of chemical synapses between sparsely labeled interneuron pairs in clusters after P14. The doubled bar-headed line represents both unidirectional and bidirectional chemical synaptic connections. The numbers of the recorded pairs are listed in the bar graph. Similar display is used in the subsequent Figures.

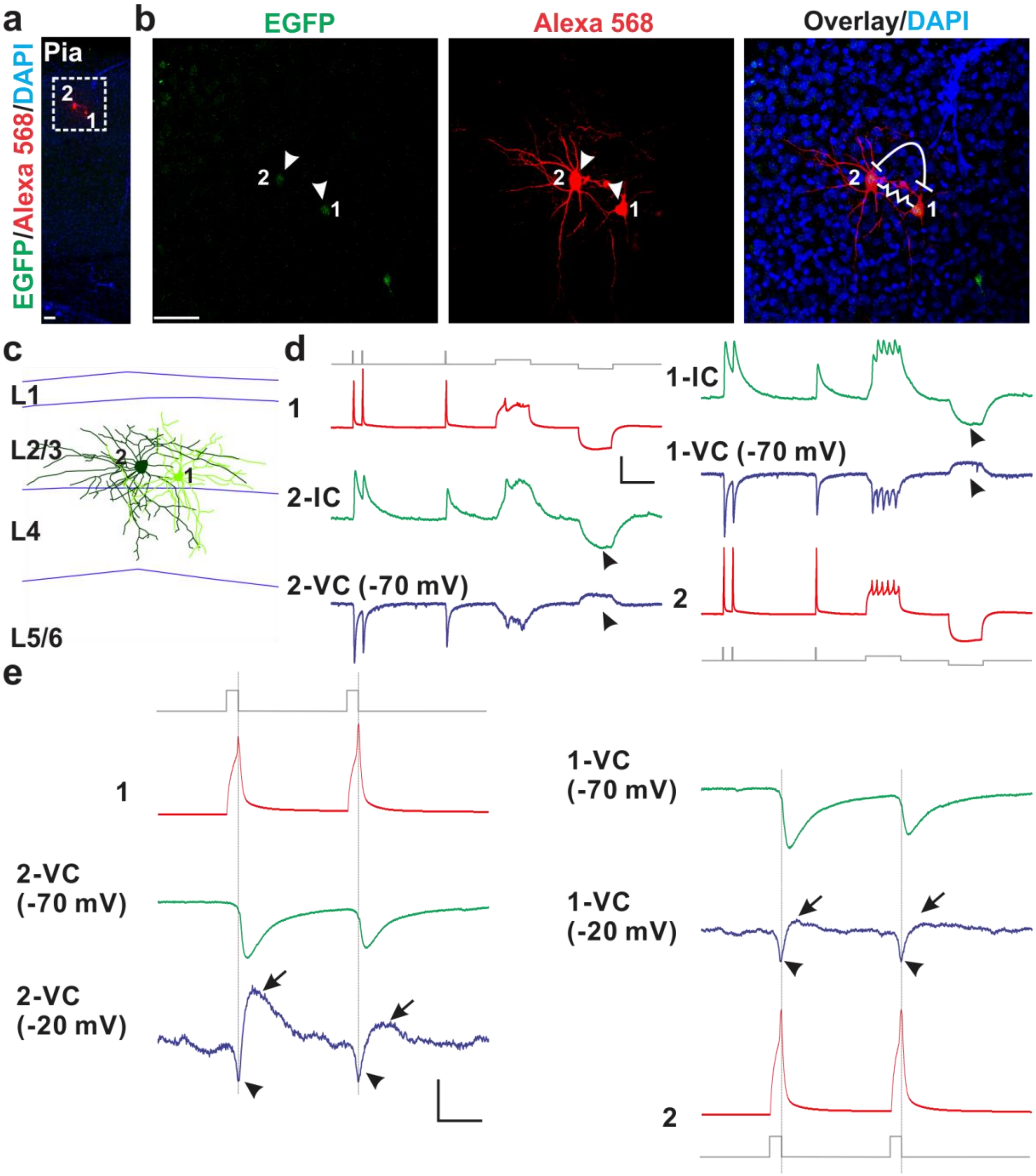

Supplementary Figure 7: Simultaneous electrical and chemical synapse formation between sparsely labeled neocortical interneurons in clusters. (a, b) Confocal images of a pair of sparsely labeled EGFP-expressing interneurons (green, 1 and 2, broken lines and arrowheads) in cluster by *in utero* RCAS injection at E12, examined by dual whole-cell patch clamp recordings at P15, and stained with DAPI (blue). Alexa 568 hydrazide (red) was included in the recording pipettes to confirm the identity of the recorded neurons. The wavy line indicates electrical coupling and the double bar-headed line indicates bidirectional chemical synaptic connections. Scale bar: 50 µm. **(c)** Morphological reconstruction of the two recorded labeled interneurons in *a*. **(d)** Sample traces of the membrane potentials or currents of the two labeled interneurons in *a* in response to brief and extended depolarizing and hyperpolarizing current injections (gray) under current-clamp (IC, green) and voltage-clamp (VC, blue) modes. Note that depolarization and hyperpolarization of one of the two EGFP-expressing labeled interneurons (red) led to

simultaneous depolarization or hyperpolarization of the other EGFP-expressing labeled interneuron (green and blue), suggesting their electrical coupling. Scale bars: 1,200 pA (gray), 50 mV (red), 3 mV (green), 30 pA (blue), and 200 msec. **(e)** Sample traces of the postsynaptic currents of the postsynaptic interneurons recorded at -70 mV (green) or -20 mV (blue) triggered by action potentials in the presynaptic interneuron (gray). Note that the postsynaptic responses become biphasic (arrowheads and arrows) at -20 mV, indicating the co-existence of both electrical and inhibitory chemical synapses. Scale bars: 1,200 pA (gray), 50 mV (red), 50 pA (green), 20 pA (blue), and 20 msec.

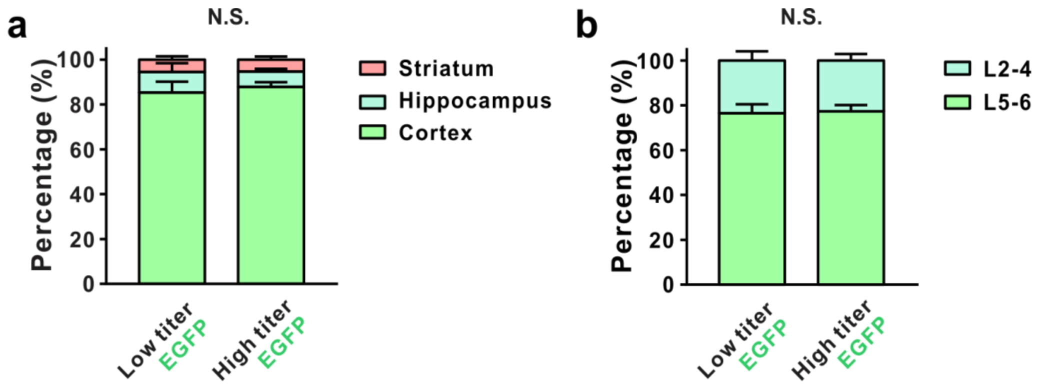

Supplementary Figure 8: Similar relative regional and layer distribution of sparsely or densely labeled interneurons originating from the MGE/PoA. (a) Quantification of the percentage of sparsely (n=6 hemispheres) or densely (n=4 hemispheres) labeled interneurons by low- or high-titer RCAS in different regions of the forebrain. N. S., not significant (unpaired t-test). **(b)** Quantification of the percentage of sparsely (n=6 hemispheres) or densely (n=4 hemispheres) labeled interneurons by low- or high-titer RCAS in different layers of the SCX and VCX. N. S., not significant (unpaired t-test).

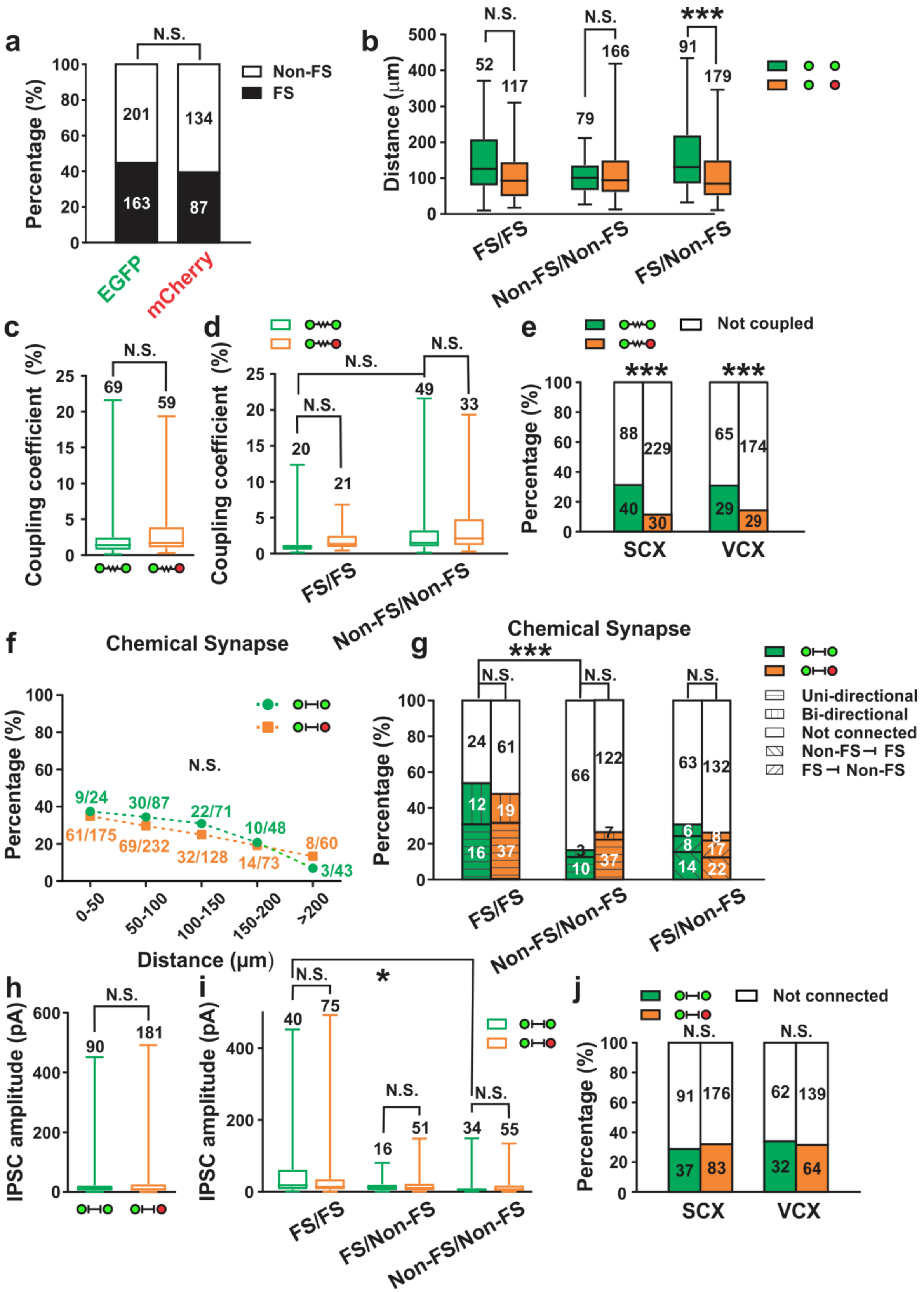
a
N.S.
Percentage (%)
Non-FS
FS
201
134
163
87
EGFP
mCherry
b
Distance (μm)
N.S.
N.S.
***
52
117
79
166
91
179
FS/FS
Non-FS/Non-FS
FS/Non-FS
c
Coupling coefficient (%)
N.S.
69
59
d
N.S.
N.S.
N.S.
20
21
49
33
e
Not coupled
Percentage (%)
***
***
88
229
65
174
40
30
29
29
SCX
VCX
f
Chemical Synapse
Percentage (%)
N.S.
9/24
30/87
22/71
10/48
3/43
61/175
69/232
32/128
14/73
8/60
0-50
50-100
100-150
150-200
>200
Distance (μm)
g
Chemical Synapse
***
N.S.
N.S.
N.S.
Uni-directional
Bi-directional
Not connected
Non-FS⊣ FS
FS⊣ Non-FS
Percentage (%)
24
61
66
122
63
132
12
19
16
37
3
7
10
37
6
8
8
17
14
22
h
IPSC amplitude (pA)
N.S.
90
181
i
IPSC amplitude (pA)
N.S.
*
40
75
N.S.
16
51
N.S.
34
55
j
Not connected
Percentage (%)
N.S.
N.S.
91
176
62
139
37
83
32
64
SCX
VCX

Supplementary Figure 9: Properties of electrical coupling and chemical synaptic connection between sparsely labeled neocortical interneurons in clusters. (a) Percentage of FS and Non-FS subtypes among recorded EGFP- and mCherry-expressing interneurons arising from the MGE/PoA after P14. The numbers of interneurons are listed in the bar graph. N.S., not significant (chi-square test). **(b)** Distances between the cell bodies of sparsely labeled (green) or non-clonally related (orange) neocortical interneuron pairs with regard to their subtypes after P14. The data are presented as the box and whisker plot with whiskers indicating the minimum and maximum values. The numbers of recorded pairs are listed above each whisker plot. ***, p<0.001; N.S., not significant (unpaired t-test). **(c)** Summary of the coupling coefficient between sparsely labeled (2.2±0.3%, n=69) and non-clonally related (2.9 ± 0.3%, n=59) neocortical interneurons. The data are presented as the box and whisker plot with whiskers indicating the minimum and maximum values. N.S., not significant (unpaired t-test). **(d)** Summary of the coupling coefficient between different subtypes. The data are presented as the box and whisker plot with whiskers indicating the minimum and maximum values. N.S., not significant (unpaired t-test). **(e)** Summary of the frequency of electrical coupling between sparsely labeled and non-clonally related interneurons in the SCX and VCX. ***, p<0.001; **, p<0.01 (chi-square test). **(f)** Summary of the frequency of chemical synaptic connectivity between sparsely labeled (green) or non-clonally related (orange) neocortical interneuron pairs with regard to the inter-soma distance after P7. N.S., not significant (chi-square test). **(g)** Summary of the frequency of chemical synaptic connectivity between sparsely labeled (green) or non-clonally related (orange) neocortical interneurons with regard to their subtypes after P14. Note the absence of preferential chemical synaptic connectivity between sparsely labeled interneurons and the significantly higher chemical synaptic connectivity between FS/FS pairs than Non-FS/Non-FS pairs. ***, p<0.001; N.S., not significant (chi-square test). **(h)** Summary of the chemical connection strength between sparsely labeled and non-clonally related neocortical interneurons. The data are presented as the box and whisker plot with whiskers indicating the minimum and maximum values. N.S., not significant. **(i)** Summary of the chemical connection strength between different subtypes. The data are presented as the box and whisker plot with whiskers indicating the minimum and maximum values. *, p<0.05; N.S., not significant. (unpaired t-test). **(j)** Summary of the frequency of chemical synaptic connectivity between sparsely labeled and non-clonally related interneurons in the SCX and VCX. N.S., not significant (chi-square test).

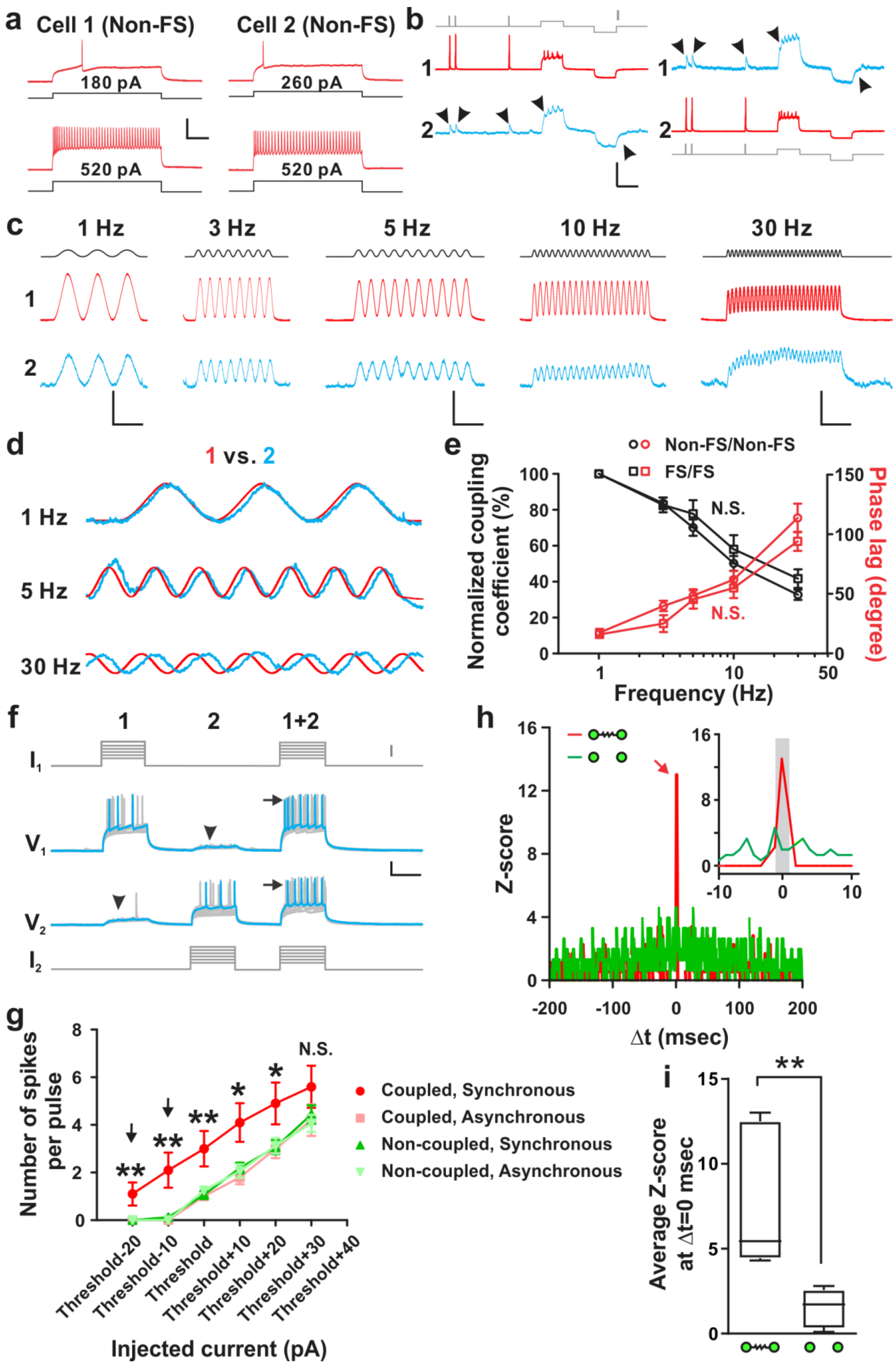

a
Cell 1 (Non-FS)
Cell 2 (Non-FS)
180 pA
260 pA
520 pA
520 pA
b
c
1 Hz
3 Hz
5 Hz
10 Hz
30 Hz
d
1 vs. 2
1 Hz
5 Hz
30 Hz
e
Non-FS/Non-FS
FS/FS
Normalized coupling coefficient (%)
Phase lag (degree)
N.S.
N.S.
Frequency (Hz)
f
1
2
1+2
h
Z-score
Δt (msec)
g
N.S.
Number of spikes per pulse
Coupled, Synchronous
Coupled, Asynchronous
Non-coupled, Synchronous
Non-coupled, Asynchronous
Threshold-20
Threshold-10
Threshold
Threshold+10
Threshold+20
Threshold+30
Threshold+40
Injected current (pA)
i
Average Z-score at Δt=0 msec

Supplementary Figure 10: Frequency-dependent signal transmission through electrical coupling between sparsely labeled neocortical interneurons in clusters promotes action potential generation and synchronous firing. (a) Firing patterns of two sparsely labeled neocortical interneurons in clusters, indicating that both are Non-FS. Scale bars: 50 mV and 200 msec. **(b)** Dual whole-cell recordings of the two sparsely labeled neocortical interneurons in *a*. Depolarizing and hyperpolarizing current injections (gray traces, scale bar, 600 pA) in one of the two sparsely labeled interneurons (driver, red) led to simultaneous depolarization or hyperpolarization of the other labeled interneuron (receiver, arrowheads, blue), indicating their electrical coupling. Scale bars: 100 mV (red), 5 mV (blue) and 200 msec. **(c)** Sample traces of the membrane potential oscillations (blue, receiver 2) produced in the electrically coupled, sparsely labeled interneurons in clusters upon injection of various frequency sine wave currents (black) into one of them (red, driver 1). Scale bars: 20 mV (red), 1.5 mV (blue) and 1 sec (1 and 3 Hz); 20 mV (red), 1.5 mV (blue) and 500 msec (5 and 10 Hz); 20 mV (red), 0.75 mV (blue) and 250 msec (30 Hz). **(e)** Normalized traces of the membrane potential oscillations between the driver (red) and receiver (blue) interneurons. **(e)** Frequency dependence of sine wave currents transmitted through electrical synapses of FS/FS pairs (square, n=5) and Non-FS/Non-FS pairs (circle, n=10). Black traces indicate the normalized coupling coefficient and red traces indicate the phase lag between the membrane potential oscillations. Note that as the frequency increases, the coupling coefficient decreases and the phase lag increases. Data are presented as mean±s.e.m. N.S., not significant (unpaired t-test). **(f)** Sample traces of synchronous (1+2) or asynchronous (1 or 2) injection of subthreshold and suprathreshold current pulses (gray) into electrically coupled, sparsely labeled neocortical interneurons in clusters. Arrowheads indicate voltage deflection due to electrical transmission. Note that synchronous injection, but not asynchronous injection, results in more action potential generation (arrows). Scale bars: 50 pA (gray), 20 mV (black), and 200 msec. **(g)** Summary of the firing rate in electrically coupled (n=10) or non-coupled (n=16) sparsely labeled interneurons in clusters responding to synchronous or asynchronous current injections. Data are presented as mean±s.e.m. *, p<0.05; **, p<0.01 (unpaired t-test). **(h)** Normalized cross-correlogram (Z-score) for an electrically coupled or a non-coupled sparsely labeled interneuron pair responding to synchronous current injections. Bin size is 1 msec. Note that the frequency of events is significantly increased around 0 msec (red arrow and inset) for electrically coupled pairs, but not for non-coupled pairs, suggesting that the preferential coupling facilitates AP generation and synchronous firing. The gray region in the inset corresponds to -1 msec $\leqslant \Delta t \leqslant 1$ msec. **(i)** Average Z score at $\Delta t = 0$ msec for electrically coupled (n=6) or non-coupled (n=8) sparsely labeled interneurons in clusters responding to synchronous current injections. The data are presented as the box and whisker plot with whiskers indicating the minimum and maximum values. **, p<0.01 (unpaired t-test).

Supplementary Table 1: Summary table showing the intrinsic membrane and firing properties of the recorded FS and Non-FS interneurons in the neocortex.

	FS (n= 243)	Non-FS (n= 292)	*p*-value (Mann-Whitney t-test)	*p*-value significance
Rest Membrane Potential (mV)	-66.5 ± 0.45	-65.6 ± 0.42	0.199	not significant
Input Resistance (MΩ)	168.3 ± 4.7	296.6 ± 8.3	10^{-15}	***
Action potential (AP)				
Threshold (mV)	-17.5 ± 0.7	-30.6 ± 0.6	10^{-15}	***
Half width (msec)	1.1 ± 0.033	2.1 ± 0.08	10^{-15}	***
Rise tau (msec)	0.9 ± 0.04	1.8 ± 0.1	10^{-15}	***
Decay tau (msec)	3.3 ± 0.2	5.8 ± 0.4	5.1×10^{-11}	***
Maximal firing frequency (Hz) (1 sec)	98.7 ± 1.8	44.0 ± 1.0	10^{-15}	***
1st 100 msec maximal firing frequency (Hz)	115.3 ± 2.5	67.6 ± 1.4	10^{-15}	***
Firing frequency adaption ratio	1.7 ± 0.03	5.4 ± 0.4	10^{-15}	***
Afterhyperpolarization (AHP)				
Time from peak (msec)	3.6 ± 0.1	11.6 ± 0.7	10^{-15}	***
Amplitude (mV)	16.6 ± 0.3	10.2 ± 0.2	10^{-15}	***

References

1. Mayer, C., *et al*. Clonally Related Forebrain Interneurons Disperse Broadly across Both Functional Areas and Structural Boundaries. *Neuron* **87**, 989-998 (2015).

2. Harwell, C.C., *et al*. Wide Dispersion and Diversity of Clonally Related Inhibitory Interneurons. *Neuron* **87**, 999-1007 (2015).
